# Overproduction of native endo-β-1,4-glucanases leads to largely enhanced biomass saccharification and bioethanol production by specific modification of cellulose features in transgenic rice

**DOI:** 10.1186/s13068-018-1351-1

**Published:** 2019-01-09

**Authors:** Jiangfeng Huang, Tao Xia, Guanhua Li, Xianliang Li, Ying Li, Yanting Wang, Youmei Wang, Yuanyuan Chen, Guosheng Xie, Feng-Wu Bai, Liangcai Peng, Lingqiang Wang

**Affiliations:** 1 Biomass and Bioenergy Research Centre, College of Plant Science and Technology, College of Life Science and Technology, Huazhong Agricultural University, Wuhan, 430070 China; 2 State Key Laboratory of Reproductive Regulation and Breeding of Grassland Livestock, School of Life Sciences, Inner Mongolia University, Hohhot, 010070 China; 3 College of Bioengineering, Jingchu University of Technology, Jingmen, 448000 China; 4 State Key Laboratory of Microbial Metabolism, School of Life Science and Biotechnology, Shanghai Jiao Tong University, Shanghai, 200240 China; 5 State Key Laboratory for Conservation and Utilization of Subtropical Agro-bioresources, College of Agriculture, Guangxi University, Nanning, 530004 China

**Keywords:** Endo-β-1,4-glucanases, Transgenic rice, GH9B, Cellulose modification, Biomass saccharification, Bioethanol production, Chemical pretreatment

## Abstract

**Background:**

Genetic modification of plant cell walls has been implemented to reduce lignocellulosic recalcitrance for biofuel production. Plant glycoside hydrolase family 9 (GH9) comprises endo-β-1,4-glucanase in plants. Few studies have examined the roles of GH9 in cell wall modification. In this study, we independently overexpressed two genes from *GH9B* subclasses (*OsGH9B1* and *OsGH9B3*) and examined cell wall features and biomass saccharification in transgenic rice plants.

**Results:**

Compared with the wild type (WT, *Nipponbare*), the *OsGH9B1* and *OsGH9B3* transgenic rice plants, respectively, contained much higher OsGH9B1 and OsGH9B3 protein levels and both proteins were observed in situ with nonspecific distribution in the plant cells. The transgenic lines exhibited significantly increased cellulase activity in vitro than the WT. The *OsGH9B1* and *OsGH9B3* transgenic plants showed a slight alteration in three wall polymer compositions (cellulose, hemicelluloses, and lignin), in their stem mechanical strength and biomass yield, but were significantly decreased in the cellulose degree of polymerization (DP) and lignocellulose crystalline index (CrI) by 21–22%. Notably, the crude cellulose substrates of the transgenic lines were more efficiently digested by cellobiohydrolase (CBHI) than those of the WT, indicating the significantly increased amounts of reducing ends of β-1,4-glucans in cellulose microfibrils. Finally, the engineered lines generated high sugar yields after mild alkali pretreatments and subsequent enzymatic hydrolysis, resulting in the high bioethanol yields obtained at 22.5% of dry matter.

**Conclusions:**

Overproduction of OsGH9B1/B3 enzymes should have specific activity in the postmodification of cellulose microfibrils. The increased reducing ends of β-1,4-glucan chains for reduced cellulose DP and CrI positively affected biomass enzymatic saccharification. Our results demonstrate a potential strategy for genetic modification of cellulose microfibrils in bioenergy crops.

**Electronic supplementary material:**

The online version of this article (10.1186/s13068-018-1351-1) contains supplementary material, which is available to authorized users.

## Background

Rice is a staple food crop around the world, providing approximately 800 million metric tons of lignocellulose-based straw annually for potential production of biofuels, feeds and chemicals [1]. Lignocellulosic ethanol is increasingly considered a partial replacement for fossil energy for the purposes of low carbon release and environmental care. The biochemical conversion of lignocellulose involves three major steps: initial physical and chemical pretreatments for wall polymer deconstruction, sequential enzymatic hydrolysis for sugar release, and final yeast fermentation for bioethanol production [2]. Due to lignocellulose recalcitrance, however, the current biomass process for bioethanol production requires a strong pretreatment and expensive enzyme loading [3]. Hence, genetic modification of plant cell walls has been raised as a promising solution to the recalcitrance in transgenic crops [4–6].

Lignocellulose recalcitrance is principally determined by the compositions, structures and interlinkages of wall polymers. Plant cell walls are composed mainly of cellulose, hemicelluloses and lignin with small amounts of pectin and wall proteins. As plant cell walls determine cell size and shape and provide mechanical support and protection against environmental stresses, genetic modification of plant cell walls may affect plant’s normal growth and mechanical strength [7]. Slightly altering cell wall composition and structure and specially improving major wall polymer properties were proposed as feasible approaches for enhanced biomass saccharification and biofuel production [4].

As the most abundant biomass on the earth, cellulose provides the largest source of fermentable glucose for bioethanol production. Cellulose is composed of β-1,4-linked glucan chains that form crystalline microfibrils by intra- and intermolecular hydrogen bonds. The crystallinity index (CrI) was characterized by a comparison of the intensities of the X-rays scattered into the reflections representing the crystalline part and into the background representing the noncrystalline part of cellulosic materials [8]. The cellulose CrI has been reported to affect biomass enzymatic saccharification negatively in various biomass residues [9–12]. In addition, the degree of polymerization (DP) of the β-1,4-linked glucans, another important cellulose feature, has also been shown to affect biomass enzymatic hydrolysis negatively [12–15]. It was reported that the conserved-site mutation of cellulose synthase 9 (OsCESA9) could reduce both cellulose DP and CrI in the rice *Osfc16* mutant plant [16]. The *Osfc16* plant displays normal growth and development, while largely enhanced biomass enzymatic saccharification and bioethanol production were achieved. The results suggest that minor alteration of cellulose features may be efficient for cell wall modification that is beneficial for biomass conversion.

Endo-β-1,4-glucanases (EGases, EC3.2.1.4) have been found in both prokaryotic and eukaryotic organisms. Plant EGases belong to subgroup E2 of glycoside hydrolase family 9 (GH9) with three subclasses (A, B, C) [17, 18]. In plants, the EGases were proposed to distinctively cleave the internal β-1,4-glycosidic bonds between two glucose moieties in the center of a polysaccharide chain [17, 19]. It is hypothesized that the cellulase from the GH9 family participates primarily in repairing or arranging cellulose microfibrils during cellulose biosynthesis in plants [20]. Among the three subclasses of GH9 family, GH9A is comprised of membrane anchored proteins, GH9B proteins are secreted with only one catalytic domain, and the GH9C class of proteins has a distinct C-terminal extended cellulose-binding domain [21, 22]. GH9A (KOR) has been characterized as an important member of the cellulose synthase complex for cellulose biosynthesis in *Arabidopsis* [23, 24]. Overexpression of the *PtCel9A1*(*PtKOR*) gene in *Arabidopsis* or overexpression of *AtKOR* in *Populus* both lead to an increase of noncrystalline cellulose level in transgenic plants [25, 26]. However, downregulation of the *KOR* gene significantly affects cellulose ultrastructure and plant growth in the poplar [25]. OsGHB1, 3 and 16 were recently proposed to have enzymatic activity for reducing cellulose crystallinity in rice plants [27, 28], but the direct genetic and biochemical evidence about their detailed roles in cellulose modification are still lacking. This would necessitate exploring postsynthesis modification of cellulose microfibrils by genetic engineering of these endogenous cellulose degradation enzymes in plants.

In this study, we showed that overexpression of two genes from the glycoside hydrolase 9B family (*OsGH9B1* and *OsGH9B3*) significantly increased reducing ends of β-1,4-glucan chains and reduced cellulose DP and CrI in transgenic rice plants. Moreover, the straw of both transgenic lines exhibited largely enhanced saccharification efficiency and increased bioethanol production after mild alkali pretreatment.

## Results

### Phylogenetic analysis and expression profiling of *OsGH9B1* and *OsGH9B3*

**Fig. 1:**
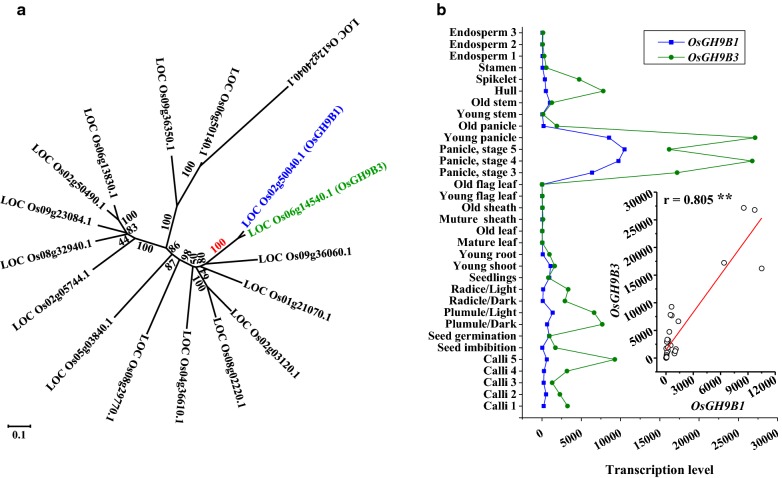
Phylogenetic analysis of GH9B family and coexpression patterns of *OsGH9B1* and *OsGH9B3*. **a** Phylogenetic tress of *OsGH9Bs*; **b** *OsGH9B1* and *OsGH9B3* coexpression profiling in all tissues covering almost entire life cycle of rice and a positive correlation between *OsGH9B1* and *OsGH9B3*; ** as significant correlation at *p* < 0.01 level (*n* = 33)

In rice, a total of 25 OsGH9s proteins were predicated as comprising the typical endo-β-1,4-glucanases (EGases, EC3.2.1.4), and these proteins have been classified into three subgroups (A, B and C) in a previous study [28]. In this study, a total of 17 GH9B members were further identified based on the phylogenetic analysis (Fig. 1a). OsGH9B1 and OsGH9B3 were closest in protein similarity among the OsGH9B members, sharing 89% amino acid identity (Fig. 1a, Additional file 1: Figure S1). Using the public expression profile data obtained from the CREP database (http://crep.ncpgr.cn) [29], we found that *OsGH9B1* and *OsGH9B3* were coexpressed with each other during the growth stages covering almost the entire life cycle of rice (r = 0.805) (Fig. 1b). In addition, both *OsGH9B1* and *OsGH9B3* genes were preferentially expressed in developing young panicles, but the expression was almost undetectable in the stem and old sheath tissues (Fig. 1b). Since rice straws are rich in secondary cell walls and have the potential to provide major lignocellulose residues for biofuels, it is of interest to explore roles of OsGH9B1 and OsGH9B3 enzymes in plant strength, cellulose modification, and biomass saccharification.

### Selection of the transgenic rice plants overproducing OsGH9B1/B3 proteins with high cellulase activity

**Fig. 2:**
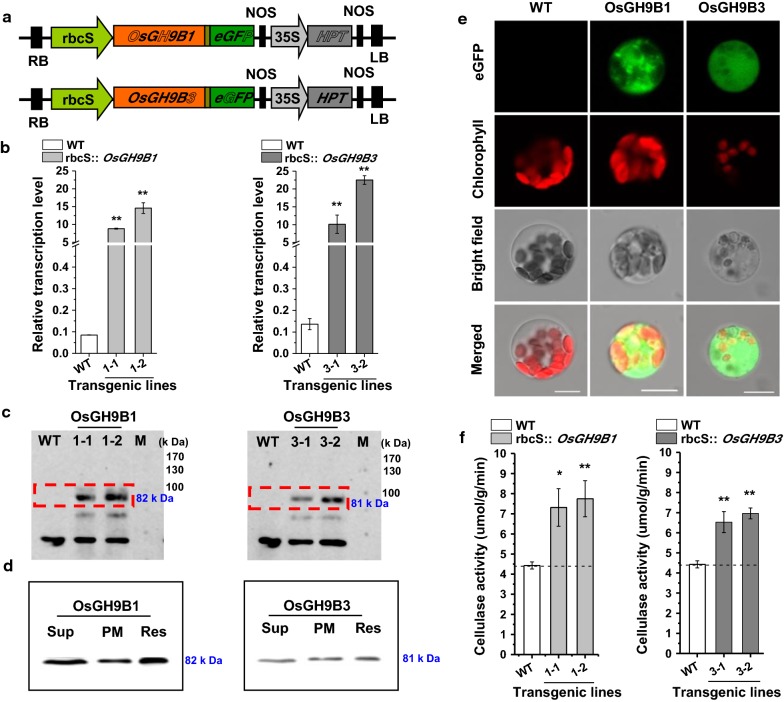
Transformation of *OsGH9B1* and *OsGH9B3* produced overexpressed proteins in the transgenic lines. **a** Constructs used for overexpression of the *OsGH9B1* and *OsGH9B3* genes. **b** Gene expression in WT (Nipponbare) plants and four transgenic lines. **c** Detection of OsGH9B1 and OsGH9B3 proteins in total proteins samples extracted from stem tissues. **d** The distribution of proteins in soluble protein samples (Sup), plasma membrane protein (PMy, and total protein samples (Res). **e** Observation of OsGH9B1/B3-eGFP in protoplast, scale bar as 10 μm. **f** Cellulase activity assay in vitro using total proteins extracted from stem tissues; Student’s *t* test performed for WT plants and transgenic lines as **p < 0.01 and *p < 0.05

To obtain transgenic rice lines, gene fragments for *OsGH9B1* and *OsGH9B3* were separately cloned into the vectors driven by green tissue-specific promoter rbcS and an eGFP tag linked to the C-terminal of the genes (Fig. 2a). Both independent transgenic lines for each of the vectors (#1-1 and #1-2 for rbcS::*OsGH9B1*, while #3-1 and #3-2 were for rbcs::*OsGH9B3*) were generated by *Agrobacterium*-mediated transformation of rice embryogenic calli (Fig. 2b). Compared with WT, the expression levels of *OsGH9B1* and *OsGH9B3* were found to be much higher in their respective transgenic lines (Fig. 2b). Western blotting analysis showed that the two independent transgenic lines from rbcS::*OsGH9B1* (#1-1 and #1-2) exhibited 82 kDa protein bands, while the other two independent lines from rbcS::*OsGH9B3* (#3-1 and #3-2) exhibited 81 kDa bands. The sizes of the two different bands corresponded to the expected sizes of OsGH9B1-eGFP and OsGH9B3-eGFP proteins, indicating that these two proteins were fully translated (Fig. 2c). Protein subcellular distribution analysis indicated that the OsGH9B1 and OsGH9B3 proteins were both located in soluble fractions and plasma membrane fractions in vitro (Fig. 2d). In addition, the fused-eGFP distribution analysis in situ indicated nonspecific distribution of fluorescence (OsGH9B1-eGFP and OsGH9B3-eGFP) in the cells of transgenic plants (Fig. 2e). Using the fluorescent cellulase assay in vitro [30], we found that all four transgenic lines showed significantly higher cellulase activities than those of the WT (Fig. 2f). Taken together, overexpression of *OsGH9B1* and *OsGH9B3* could largely increase their protein levels and significantly enhance cellulase activities in the transgenic plants.

### Largely enhanced biomass saccharification and bioethanol production in the *OsGH9B1/B3* transgenic plants

**Fig. 3:**
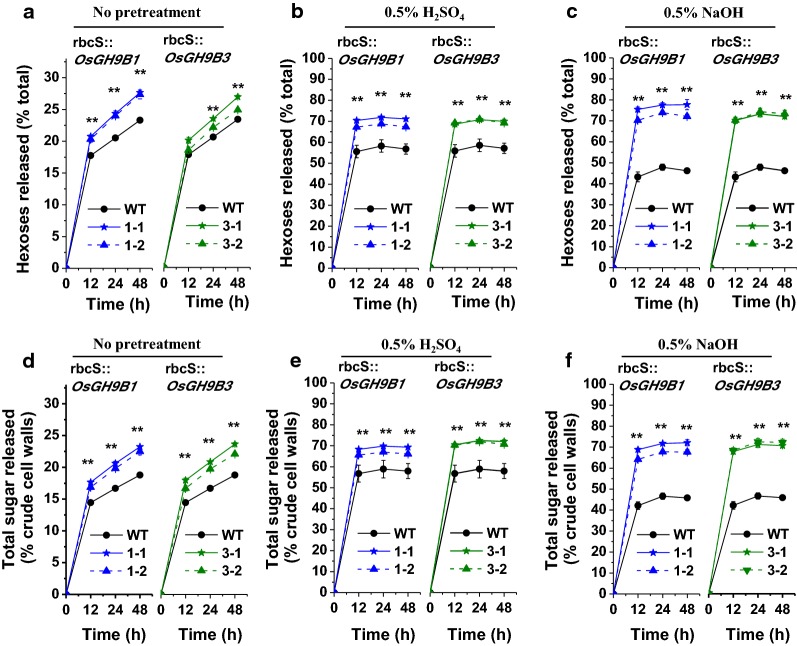
Biomass enzymatic saccharification in the *OsGH9B1* and *OsGH9B3* transgenic lines. **a**–**c** Hexoses yields released from enzymatic hydrolysis (% of total dry mass) without pretreatment, 0.5% H_2_SO_4_ pretreatment, and 0.5% NaOH pretreatment, respectively. **d**–**f** Total sugars yields released from pretreatment (if any) and subsequently enzyme hydrolysis (% of crude cell walls) without pretreatment, 0.5% H_2_SO_4_ pretreatment, and 0.5% NaOH pretreatment, respectively. Student’s *t* test performed for WT and transgenic plants as **p < 0.01

To characterize biomass enzymatic saccharification (digestibility), this study measured both hexoses (% total hexoses in crude cell walls) and total sugar yields (% crude cell walls) released from commercial mixed-cellulase enzymatic hydrolysis (Fig. 3). Without any pretreatment, all *OsGH9B1* and *OsGH9B3* transgenic lines exhibited significantly enhanced hexoses and total sugar yields after enzymatic hydrolysis, compared with WT (Fig. 3a, d). Under 0.5% H_2_SO_4_ pretreatments, the *OsGH9B1* and *OsGH9B3* transgenic lines had much higher hexoses and total sugar yields than those of the WT (Fig. 3b, e). Notably, upon 0.5% NaOH pretreatment, the transgenic lines had either yielded more than 70% hexoses or the total sugar yields of close to 68% after 12 h enzymatic hydrolysis, whereas those of WT were both less than 43% (Fig. 3c, f). Hence, these results clearly demonstrate that the *OsGH9B1* and *OsGH9B3* transgenic rice lines showed a consistently enhanced biomass enzymatic saccharification.

**Fig. 4:**
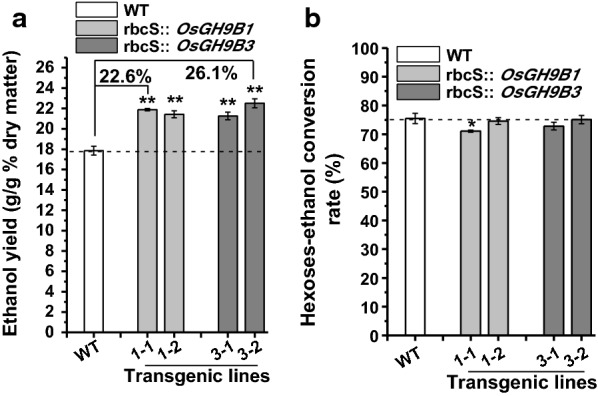
Bioethanol productivity by yeast fermentation using total sugars released from enzymatic hydrolysis after 0.5% NaOH pretreatment as substrates in the transgenic lines. **a** Bioethanol yields (g/g % dry matter). **b** Sugar–ethanol conversion rates. Student’s *t*-test performed for WT and transgenic plants as **p < 0.01

Given that the mild alkali pretreatment is superior to acid pretreatment for higher hexose yields in this study, 0.5% NaOH pretreatment of rice straw was used for subsequent enzymatic hydrolysis and final yeast fermentation into bioethanol production (Fig. 4). By comparison, the transgenic lines exhibited 21–26% ethanol yield per gram of dry matter higher than the yield of the WT (Fig. 4a). However, all transgenic lines and WT showed a similar sugar–ethanol conversion rate at 75% (Fig. 4b). Notably, the ethanol yields obtained from *OsGH9B1* to *OsGH9B3* transgenic lines could reach 21.9% and 22.5% (g/g, % dry matter), respectively, much higher than those in previous studies (Table 1).

**Table 1:** Bioethanol production in rice

Pretreatments	Ethanol production (% dry biomass)	References
rbcS::*OsGH9B1* 0.5% NaOH 50 °C for 2 h + 1% tween-80	21.9	This study
rbcS::*OsGH9B3* 0.5% NaOH 50 °C for 2 h + 1% tween-80	22.5	This study
1% H_2_SO_4_ 121 °C for 15 min + ultrasound: 40 W 50 °C for 10 min	11	[61]
0.65% HNO_3_, 158.8 °C for 5.86 min	14.5	[62]
Torrefaction 220 °C for 40 min	15	[63]
1% maleic acid 190 °C for 3 min	16.9	[64]
Popping pretreatment (dry sample 20 °C/min to 220 °C, 1.96 MPa)	17.2	[65]
2% Lime 120 °C for 60 min + CO_2_ neutralization	19.1	[66]
Transgenic plant: 1% sodium hydroxide + 1% Tween-80	21	[67]
21% aqueous-ammonia 69 °C for 10 h + initial loading 3% glucan	21.1	[68]

### Slightly affected plant growth and unaltered mechanical strength in transgenic plants

**Fig. 5:**
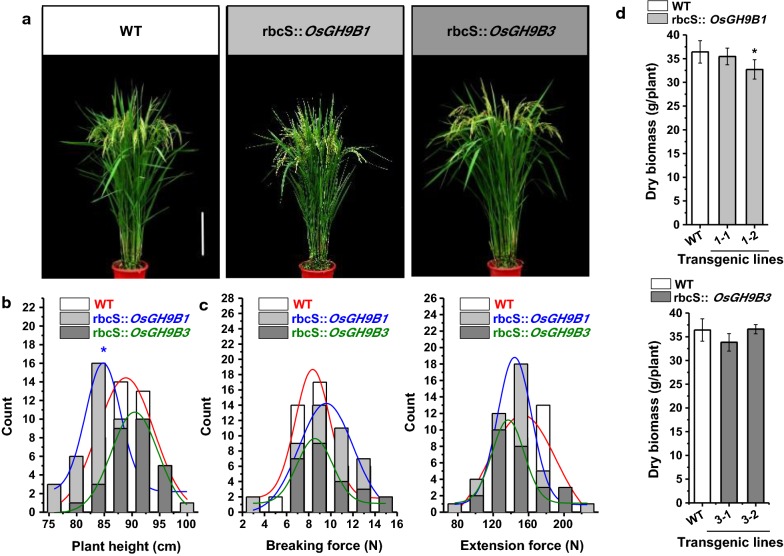
Phenotypes and mechanical strengths of the *OsGH9B1* and *OsGH9B3* transgenic plants. **a** Plant growths of representative transgenic plants and WT at filling stage, scale bar as 20 cm. **b** Plant height (WT: *n* = 46, *OsGH9B1*: *n* = 43, *OsGH9B3*: *n* = 28). **c** Breaking force (WT: *n* = 45, *OsGH9B1*: *n* = 43, *OsGH9B3*: *n* = 27) and extension force (WT: *n* = 38, *OsGH9B1*: *n* = 41, *OsGH9B3*: *n* = 26) of the stem tissues. **d** Dry biomass. Student’s *t*-test performed for WT and transgenic plants as **p < 0.01 and *p < 0.05

In the 2-year field experiments, we observed small changes in plant growth and development in all transgenic lines, with the two representative plants shown in Fig. 5a. Compared with WT, the *OsGH9B1* transgenic plants were slightly shorter, while the plant height of the *OsGH9B3* transgenic lines remained unchanged (Fig. 5b). The breaking force and extension force are two major typical parameters accounting for plant mechanical strength [31–33]. The breaking and extension forces of *OsGH9B1* and *OsGH9B3* transgenic plants were found to be similar to those of WT (Fig. 5c). Meanwhile, the total dry weight of the transgenic rice plants showed no significant difference from the WT, except the line #1-2 of *OsGH9B1* transgenic plant showed a slight reduction (Fig. 5d). Hence, overexpressions of *OsGH9B1* and *OsGH9B3* only resulted in a slight impact on plant growth and mechanical strength in the transgenic rice plants.

### Small impact on cell wall contents and morphology in transgenic lines

**Fig. 6:**
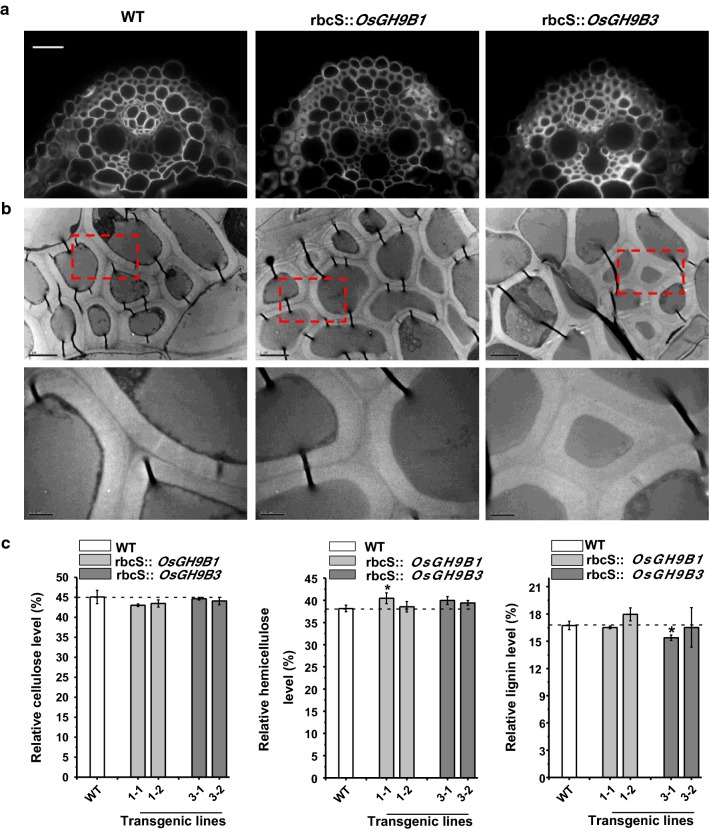
Cell wall morphologies and compositions in the *OsGH9B1* and *OsGH9B3* transgenic lines. **a** Sclerenchyma cells with Calcofluor White staining observed under a fluorescence microscopy, scale bar as 50 μm. **b** Cell walls morphology of sclerenchyma cells under transmission electron microscopy, scale bar as 2 μm (up) and 0.5 μm (down). **c** The contents of three major cell wall polymers (% total). Student’s *t* test performed for WT and transgenic plants as *p < 0.05

Using calcofluor white staining with the vein tissues at the seedling stage, the representative transgenic lines of *OsGH9B1* and *OsGH9B3* exhibited typical cell wall morphology similar to the cell wall morphology of WT (Fig. 6a). Meanwhile, this study applied transmission electron microscopy to observe primary and secondary cell walls of the sclerenchyma cells. Again, the wall morphology was not altered in the transgenic lines (Fig. 6b).

Furthermore, this study determined the contents of three major cell wall polymers of the mature stem tissues in the transgenic rice plants. Compared with WT, the *OsGH9B1* transgenic line #1-2 showed similar levels of all three wall polymers, whereas line #1-1 showed a moderate difference in the content of hemicelluloses. By comparison, the *OsGH9B3* transgenic line #3-1 had a relatively lower lignin level than the WT by 8%, whereas the line #3-2 had three wall polymer contents similar to the WT (Fig. 6c). Taken together, overexpressions of *OsGH9B1* and *OsGH9B3* genes had little impact on wall polymer content and morphology in the transgenic plants, consistent with the observations of the normal mechanical strength detected in the transgenic plants (Fig. 6).

### Remarkably reduced cellulose DP and CrI and increased CBHI enzymatic hydrolysis of cellulose substrate

**Fig. 7:**
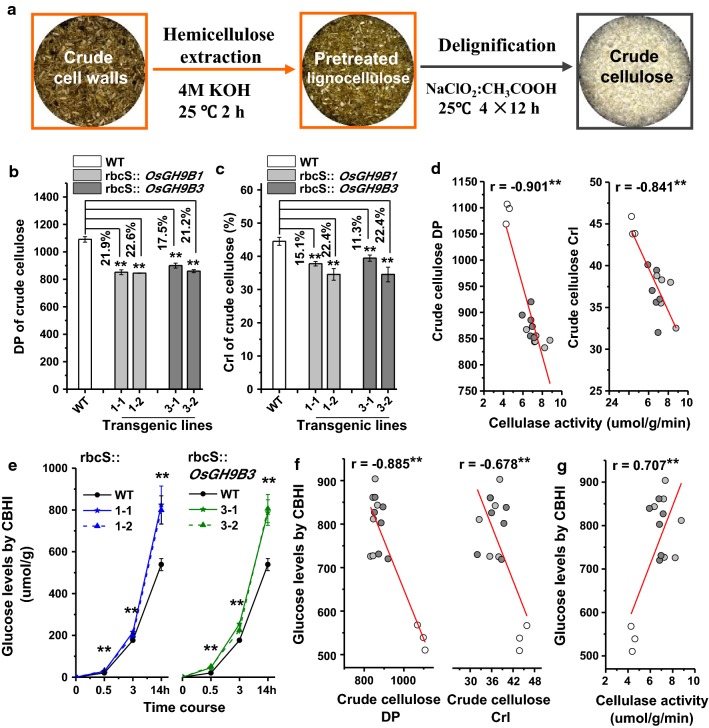
Cellulose DP and CrI in the *OsGH9B1* and *OsGH9B3* transgenic lines. **a** Schematic flow of crude cellulose extraction. **b** Crude cellulose DP. **c** Crude cellulose CrI. **d** Correlation of cellulase activity and cellulose CrI and DP (*n* = 15). **e** Glucose yield of cellobiose CBHI hydrolyzes using crude cellulose as samples in three time points. **f** Correlation of cellulose features (DP, CrI) and the glucose levels after CBHI hydrolysis (*n* = 15). **g** Correlation between cellulase activity and the glucose levels by CBHI hydrolysis (*n* = 15). Different grayscale circle in **d**, **f** and **g** indicate the biological data from WT (White), *OsGH9B1* (Light gray), and *OsGH9B3* (Dark gray), respectively

This study detected degree of polymerization (DP) of β-1,4-glucans and cellulose crystallinity index (CrI) in the transgenic plants using the crude cellulose samples after removal of hemicellulose and lignin (Fig. 7a). All *OsGH9B1* and *OsGH9B3* transgenic lines showed significantly reduced cellulose DP by 17–23%, compared with WT (Fig. 7b). Meanwhile, cellulose CrI was decreased by 11–22% in transgenic lines (Fig. 7c). Furthermore, those two cellulose parameters (DP and CrI) were found to be negatively correlated with the cellulase activities in the transgenic plants with the r values at -0.901 and -0.841, respectively (*n* = 15) (Fig. 7d), suggesting the potential roles of OsGH9B1 and OsGH9B3 proteins in determining cellulose features.

Moreover, using GC/MS analysis, this study investigated the dynamic profiling of cellobiose releases (calculated as glucose releases) from crude cellulose of the mature stem tissues during cellobiohydrolase (CBHI; E.C. 3.2.1.91) hydrolysis. All *OsGH9B1* and *OsGH9B3* transgenic lines showed much higher glucose yields than those of the WT in three time points during CBHI hydrolysis (Fig. 7e). The glucose yields of CBHI hydrolysis were significantly and negatively correlated with both DP and CrI of crude cellulose (*n* = 15) (Fig. 7f). Since the CBHI enzyme has been known to specifically attack the reducing ends of β-1,4-glucan chains, the increased glucose yield is most likely due to the increased short β-1,4-glucan chains in the transgenic plants, which were accountable for the reduction of cellulose DP and CrI. More important, the glucose yields of CBHI hydrolysis were significantly and positively correlated with the cellulase activities detected in the stem of the transgenic pants with r value at − 0.707 (*n* = 15) (Fig. 7g). Therefore, the multiple results suggested that the OsGH9B1 and OsGH9B3 proteins should have cellulase activities for the modification of cellulose microfibrils in the transgenic plants.

### Mechanism of the overproduced OsGH9B1 and OsGH9B3 for enhancing lignocellulose saccharification and ethanol production

**Fig. 8:**
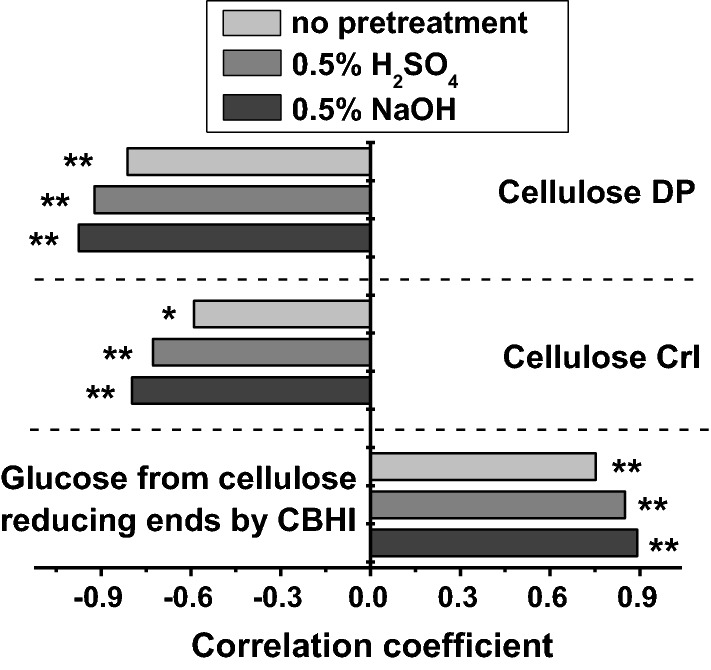
Correlation analysis between enzymatic saccharification and cellulose features. * and ** Indicate the significant differences at *p* < 0.05 and *p* < 0.01, respectively (*n* = 15)

To understand the mechanism that the overexpressed OsGH9B1 and OsGH9B3 enhanced both lignocellulose saccharification and the subsequent bioethanol production, we performed a correlation analysis between biomass saccharification efficiency and cellulose features. Both cellulose DP and CrI values were negatively correlated with the hexose yields released from enzymatic hydrolysis either with or without pretreatments at *p* < 0.01 and 0.05 levels (*n* = 15) (Fig. 8), consistent with our previous findings that cellulose DP and CrI are the key parameters that negatively affect lignocellulose enzymatic hydrolysis in various plant species examined [12, 34–40]. Moreover, this study found that the glucose released by CBHI hydrolysis from the cellulose reducing ends was positively correlated with lignocellulose saccharification under various conditions (*p* < 0.01, *n* = 15) (Fig. 8). It is rational that the increased number of reducing ends in cellulose chains could fundamentally enhance the biomass saccharification in the transgenic plants. Taken together, we concluded that the increased reducing ends of the cellulose microfibrils, consistent with the reduced cellulose DP and CrI, should be the major causes for the largely enhanced biomass saccharification and bioethanol production in the transgenic plants.

## Discussion

Genetic modification of plant cell walls has been implicated in the largely enhanced lignocellulose enzymatic saccharification and biofuel production in transgenic crops. However, because plant cell walls have extremely complicated structures and diverse biological functions, large modifications of cell walls may affect plant growth and development. Hence, the selected transgenic crops should not only have largely increased biomass saccharification, but also need to maintain a normal plant growth and mechanical strength. Over the past years, attempts have been made to enhance lignocellulose enzymatic hydrolysis by altering hemicellulose features or reducing lignin contents [37, 38, 41–46], but most of the transgenic plants displayed defects in growth and strength or limited enhancement of biomass saccharification. This study has indicated that the minor modifications of cellulose microfibrils could have a large impact on the enhancement of biomass enzymatic hydrolysis with only slightly altered plant growth by overexpressing *OsGH9B1/B3* genes in the transgenic rice crops, providing a powerful genetic engineering strategy for selection of bioenergy rice and other energy crops.

**Fig. 9:**
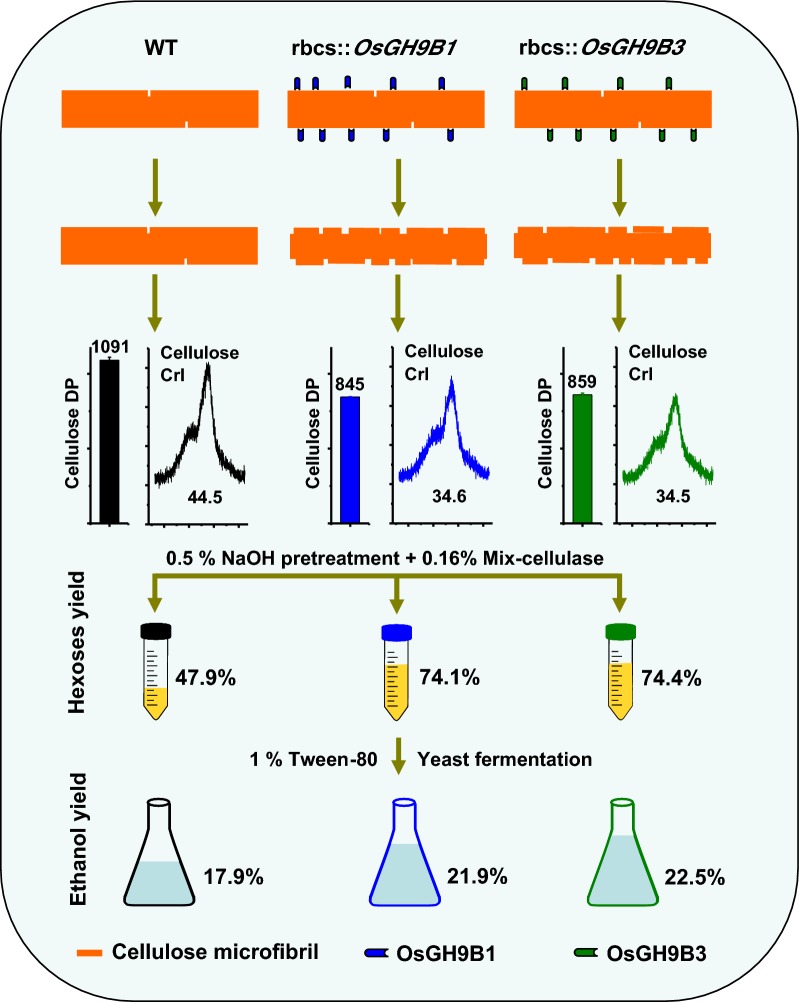
Hypothetical model for the involvement of OsGH9B1 and OsGH9B3 in postmodification of cellulose microfibrils. Expression of OsGH9B1 and OsGH9B3 proteins remarkably increased the reducing ends of cellulose microfibrils, causing the reduced DP and CrI of cellulose, leading to the largely enhanced biomass saccharification and bioethanol production in the transgenic plants

Although the members of the OsGH9B subclass have been proposed to have cellulase activities in rice [27, 28], their roles in plant cell wall remodeling remain largely unknown. We generated overexpressing OsGH9B1 or OsGH9B3 transgenic lines and investigated their abundance, subcellular distribution, and cellulase activity. We then systematically conducted an analysis of plant phenotypes, mechanical strength, biomass yield, polymer content, wall morphology, reducing ends of cellulose, DP and CrI of crude cellulose. Furthermore, the sugar yields and subsequent ethanol yield after pretreatments and bioprocessing were determined. These results together with association analysis allowed us to get a comprehensive understanding of the genetic and biochemical function of the OsGH9B1 or OsGH9B3 in cell wall modification. The major conclusions of this study were (1) the transgenic lines OsGH9B1/B3 consistently exhibited high cellulase enzymatic activity and improved cellulose features and biomass digestibility with little impact on the entire cell walls and mechanical strength, indicating the modification was specific in cellulose microfibrils; (2) the remarkable changes in reducing ends of β-1,4-glucan chains, cellulose DP and CrI were probably due to the cleavage of cellulose microfibrils (post modification), as we found that the mutation of cellulose synthase 9 will decrease both cellulose level and mechanical strength in addition to the reduced DP and CrI due to early termination of β-1,4-glucan chain elongation [16]. Taken together, this study proposed a model highlighting that the overproductions of OsGH9B1 and OsGH9B3 enzymes could increase reducing ends in the β-1,4-glucan chain, leading to largely reduced cellulose DP and CrI probably by specific postmodification of cellulose microfibrils in the transgenic rice plants (Fig. 9).

The cellulose DP and CrI are the key parameters that are negatively associated with lignocellulose enzymatic hydrolysis under various pretreatments in many biomass residues examined [9, 10, 12–15, 34, 35, 37–39]. Although the cellulose CrI value is in part associated with cellulose DP, it is also affected by the levels of wall polymers and their crosslinks [11, 40, 47, 48]. However, the finding that there was little change in the contents of three major wall polymers implied that reduction of cellulose CrI was due to the decreased DP in OsGH9B1/B3 transgenic lines in this study.

One of the advantages of this study is that we independently introduced two members of the *OsGH9B* gene family (*OsGH9B1* and *OsGH9B3*) into rice plants. The transgenic lines exhibited similar alterations in cellulose properties, indicating the similar roles of *OsGH9B1* and *OsGH9B3* in postmodification of cellulose microfibrils. In *Arabidopsis* and *Populus*, several genes have been identified to be close to *OsGH9B1* and *OsGH9B3* based on phylogenetic analysis of the GH9 family in a previous study [49], yet their functions remain to be explored. It is of interest to test whether these members have similar cellulase activities specific for cellulose modification and for genetic improvement of biofuel plants in the future.

## Experimental procedures

### Phylogenetic analysis

The Neighbor-Joining method was used for phylogenetic tree analysis [50], and the optimal tree was plotted with the sum of branch length at 4.47282155. The tree is drawn to scale, with branch lengths in the same units as those of the evolutionary distances used to infer the phylogenetic tree. The evolutionary distances were computed using the Poisson correction method [51] in the units of the number of amino acid substitutions per site. The phylogenetic analysis involves a total of 17 amino acid sequences, and all positions containing gaps and missing data were eliminated. Evolutionary analyses were conducted in MEGA6 [52].

### Plasmid vector construction and transgenic line selection

The full-length cDNA of *OsGH9B1* and *OsGH9B3* were, respectively, amplified from young panicle of rice cultivar “*Nipponbare*” (a *japonica* variety) using primers as shown in Additional file 1: Table S1. *OsGH9B1* and *OsGH9B3* were separately inserted into the modified plant binary vector pCAMBIAI1300 driven by the rubisco small subunit (rbcS) promoter. Meanwhile, the *eGFP* gene was constructed to fuse with C-terminal of these genes. These two constructs were independently transferred into “Nipponbare” by *Agrobacterium*-mediated transformation with the “EHA105” strain. The homozygous transgenic lines were identified based on the hygromycin B screening for 3-4 generations, double checked by PCR analysis and verified by qRT-PCR and Western analysis as described below.

### Total RNA isolation and qRT-PCR analysis

Total RNA was extracted from the second internodes of stem tissues at heading stage using the Trizol reagent (Invitrogen). cDNA was synthesized with the GoScript™ Reverse Transcription System (Promega, USA). Quantitative real time-PCR (qRT-PCR) was performed in triplicate using the SYBR Green PCR Master Mixture (ZF101, ZOMANBIO). A rice polyubiquitin gene (OsUBQ1) was used as the internal control. All primers used for qRT-PCR were listed in Additional file 1: Table S1.

### Protein preparation and Western blot analysis

Total proteins were collected from the supernatants extracted from the second internodes of stem tissues at heading stage. The proteins were centrifuged at 100,000*g* for 1 h at 4 °C to collect the residues as the plasma membrane proteins as described by Li et al. [16]. The supernatants were precipitated with acetone (3:1, v/v) at − 20 °C for 12 h to collect soluble proteins. The protein samples were loaded into 12% SDS-PAGE gel for protein separation, and the Western blot analysis was conducted as described by Li et al. [16]. The commercial GFP antibody was used as the primary antibody reaction at 1:1000 dilutions and the affinity-purified phosphatase-labeled goat antirabbit IgG was applied as secondary antibody reaction at 1:5000. Protein bands were detected by the ECL Plus Western Blotting Detection, and scanned under a GeneGnome XRQ (Syngene Inc., Maryland, US).

### GFP fluorescence observation

The protoplasts were obtained from the stem tissues of 2-week-old seedlings of rice as described by Yoo et al. [53]. CLSM imaging via a Leica TCS SP8 confocal laser scanning system (Leica Microsystems, Wetzlar, Germany) was performed for GFP fluorescence observation.

### Cellulase activity assay in vitro

The second internodes of rice stem tissues were ground into the powders by liquid nitrogen, and extracted with 100 mM sodium acetate trihydrate buffer (pH 5.5). After centrifugation at 12,000*g* for 10 min at 4 °C, the supernatants were collected for cellulase activity assay as described by Dai et al. [30]. A total of 50 μL supernatant proteins were incubated with 50 μL 0.5 mM 4-methylumbelliferyl β-D-cellobioside (Sigma-Aldrich, USA) at 55 °C for 30 min and stopped by adding 30 μL 0.2 M sodium carbonate. The fluorescence intensity was recorded using a Spectrum Microplate Spectrophotometer (Tecan Infinite M200 PRO, Switzerland) at the excitation wavelength of 365 nm and emission wavelength of 480 nm. The protein concentration was determined by Bradford method [54] using BSA as the standard. All cellulase activity assays were independently conducted in triplicate.

### Determination of the mechanical strength in the rice stem

The breaking and extension forces were detected in the stem tissues at the milk maturity stages of rice as described by [34, 35]. Totally 45, 43 and 27 individual plants of WT, transgenic lines OsGH9B1 and OsGH9B3 were, respectively, used for breaking forces examination. Total 38, 41 and 26 individual plants of WT, transgenic lines OsGH9B1 and OsGH9B3 were measured for extension forces, respectively.

### Fluorescence microscopy and transmission electron microscopy analyses

The third leaf veins of three-leaved old seedlings were used for observation of cell wall morphology under fluorescence microscopy and transmission electron microscopy.

For fluorescence microscopic observation, the samples were fixed with 4% (w/v) paraformaldehyde, and dehydrated through an ethanol gradient (30%, 50%, 70%, 90% and 100%, each for 30 min), and then embedded in the paraplast plus. The sections (of 8 μm thickness) were cut using a microtome (RM2265 Leica Microsystems, Leica, Nussloch, Germany) and placed on lysine-treated slides which were dried for 2 days at 37 °C, and dewaxed with xylene and hydrated through an ethanol series (100–0%). The sections were stained with calcofluor white fluorochrome (Calcofluor White Stain; Fluka), and imaged using a microscope (Olympus BX-61, Olympus, Tokyo, Japan). For transmission electron microscopic observation, the samples were prepared as previously described by Fan et al. [34, 35]. The samples were postfixed in 2% (w/v) OsO4 for 1 h after extensively washing in the PBS buffer and embedded with Suprr Kit (Sigma-Aldrich, St. Louis, MO, USA). Sample sections were cut with an Ultracut E ultrami-crotome (Leica) and picked up on formvar-coated copper grids. After poststaining with uranyl acetate and lead citrate, the specimen was viewed under a Hitachi H7650 (Hitachi Ltd., Tokyo, Japan) transmission electron microscope.

### Extraction of crude cell walls and cellulose samples

The homozygous transgenic rice plants and the wild type (*Nipponbare*) were grown in the experimental field of Huazhong Agricultural University, Wuhan, China. The mature stem tissues were dried, ground into powder through 40 mesh (0.425 mm × 0.425 mm) , and stored in a dry container. The powder samples were extracted with ddH_2_O at 25 °C for 2 h to remove soluble sugar, followed by chloroform: methanol (1:1) at 25 °C for 1 h and then methanol to remove lipids. The residues were then extracted with 70% (v/v) ethanol at 25 °C for 12 h, and washed two times with 70% (v/v) ethanol. The remaining residues were washed two times with acetone and dried under vacuum to obtain the final crude cell wall sample. The crude cell walls were further extracted with 4 M KOH (containing 1.0 mg/mL sodium borohydride) at 25 °C for 2 h to remove hemicellulose, and the remaining pellet was washed five times with ddH_2_O and extracted with 8% (w/v) sodium chlorate (containing 1.5% acetic acid, v/v) at 25 °C for 48 h to remove lignin. The remaining pellet was washed six to eight times with ddH_2_O and dried under vacuum to obtain the final crude cellulose sample.

### Hemicellulose and cellulose extraction and determination

The crude cell wall samples from the above described extraction, were used for further extraction of hemicelluloses and cellulose fractions as described by Fan et al. [34, 35]. Total hexoses and pentoses released from 4 M KOH extraction and the pentoses released from 67% (v/v) H_2_SO_4_ hydrolysis were summed for determining the total hemicellulose content. Cellulose was estimated by calculating total hexoses from 67% (v/v) H_2_SO_4_ hydrolysis. The anthrone/H_2_SO_4_ method [55] and orcinol/HCl method [56] were applied for the hexoses and pentoses assay. d-glucose and d-xylose were prepared to plot standard curves, and the deduction from pentoses reading at 660 nm was carried out for calculation of final hexoses in order to eliminate the interference of pentose on hexose reading at 620 nm.

### Lignin determination

Total lignin was assayed using a two-step acid hydrolysis method according to the Laboratory Analytical Procedure of the National Renewable Energy Laboratory [57]. The crude cell wall samples were hydrolyzed with 67% (v/v) H_2_SO_4_ at 25 °C for 90 min with a gentle shaking at 115 rpm, and subsequently diluted to 3.97% (w/w) with distilled water and heated at 115 °C for 60 min. The supernatant liquids were read at 205 nm for acid soluble lignin, and the remaining residues were placed in a muffle furnace at 575 °C ± 25 °C for 4 h for the acid insoluble lignin assay.

#### Detection of cellulose features (DP, CrI)

The crude cellulose DP assay was performed using viscosity method as previously described by Zhang et al. [12]. Cellulose CrI was detected using X-ray diffraction (XRD) method (Rigaku-D/MAX instrument, Uitima III, Japan) as described by Segal et al. [58].

### Crude cellulose hydrolysis by β-1,4-exoglucanase (cellobiohydrolase-CBHI)

CBHI enzyme hydrolysis assay was performed using crude cellulose samples. The crude cellulose samples (10 mg) were incubated with 0.5 U CBHI (E.C. 3.2.1.91; Megazyme, USA) at 50 °C for a time course of reactions at 30 min, 3 h, and 14 h. After centrifugation at 3000*g*, the supernatants were collected and treated with 2 M TFA at 120 °C for 1 h, and *Myo*-inositol (20 μg) was added as the internal standard. The supernatants were then dried under vacuum to remove TFA. Distilled water (200 μL) and freshly prepared solution of sodium borohydride (100 μL, 100 mg/mL in 6.5 M aqueous NH_3_) were added to each sample, incubated at 40 °C for 1 h, and the excess sodium borohydride was decomposed by adding acetic acid (200 μL). The sample was transferred into a 25-mL glass tube, and 1-methylimidazole (600 μL) and the acetic anhydride (4 mL) were added and mixed well to perform an acetylation reaction at 25 °C for 30 min. The excess acetic anhydride was decomposed by adding distilled water (10 mL). Dichloromethane (3 mL) was added, mixed gently, and left standing for phase separation. The collected lower phase was dehydrated by adding anhydrous sodium sulfate and analyzed using GC–MS (SHIMADZU GCMS-QP2010 Plus) as described by Li et al. [47].

### Chemical pretreatments and biomass enzymatic saccharification

Alkali pretreatment: The crude cell wall samples were incubated with 6 mL 0.5% NaOH (w/v) and shaken at 150 rpm for 2 h at 50 °C. After centrifugation at 3000*g* for 5 min, the supernatants were collected for determination of hexoses and pentoses released from alkali pretreatment. The remaining pellets were subsequently washed five times with 10 mL of distilled water for sequential enzymatic hydrolysis.

Acid pretreatment: The crude cell wall samples were incubated with 6 mL of 0.5% H_2_SO_4_ (v/v) at 121 °C for 20 min in an autoclave (15 psi). The samples in tubes were then shaken at 150 rpm for 2 h at 50 °C. After centrifugation at 3000*g* for 5 min, the supernatants were collected for the determination of hexoses and pentoses released from the acid pretreatment, and the pellets were washed five times with 10 mL of distilled water for sequential enzymatic hydrolysis.

Enzymatic hydrolysis: The remaining residues obtained from alkali or acid pretreatment were washed with 6 mL of mixed-cellulase reaction buffer (0.2 M acetic acid–sodium acetate, pH 4.8), then incubated with 0.16% (w/v) mixed cellulases (Imperial Jade Biotechnology Co., Ltd. Ningxia 750002, China) with the final concentrations of cellulase at 10.60 FPU/g biomass, and xylanase at 6.72 U/g biomass. The measurement of mixed-cellulase activity was based on the filter paper assay according to the International Union of Pure and Applied Chemistry (IUPAC) guidelines, 1 FPU = 1 μmol/min of glucose formed during the hydrolysis reaction. The measurement of xylanase activity used 1% (w/v) xylan (Sigma-Aldrich Co. LLC, California, USA), as the substrate, 1 U = 1 μmol/min of xylose, formed during the hydrolysis reaction. The samples were shaken at 150 rpm at 50 °C with a time course hydrolysis for 12 h, 24 h, and 48 h. After centrifugation at 3000*g* for 10 min, the supernatants were collected for hexoses and pentoses assay.

### Yeast fermentation and bioethanol measurement

The biomass powders were incubated with 6 mL 0.5% NaOH (w/v), shaken at 150 r/min for 2 h at 50 °C. After pretreatments, the biomass residues and supernatants were neutralized to pH 4.8 using appropriate amounts of H_2_SO_4_. Then, mixed cellulases were added to the final enzyme concentration at 1.6 g/L cosupplied with 1% Tween-80, and incubated at 150 rpm for 48 h at 50 °C. After enzymatic hydrolysis, the supernatants were collected for yeast fermentation. Yeast fermentation and ethanol measurement were performed as previously described by Jin et al. [59] and Zahoor et al. [60] by means of *Saccharomyces cerevisiae* (Angel yeast Co., Ltd., Yichang, China) and the dichromate oxidation method.

### Data collection and statistical analysis

Biological triplicate samples were collected for each transgenic line selection, and chemical analysis was performed in technical triplicates. The SPSS statistical software was used for data analysis. Prior to statistical analysis, all data were analyzed by a Kolmogorov–Smirnov test to check for normal distribution of samples. Pearson correlation analysis was performed for correlation coefficients calculation, and Student’s *t* test was used for comparison analysis.

## Additional file

**Additional file 1: Figure S1.** Alignment between OsGH9B1 and OsGH9B3.** Table S1.** The primers used for gene cloning and expression analysis in this study.
