# Supplementary material for: Overproduction of native endo-β-1,4-glucanases leads to largely enhanced biomass saccharification and bioethanol production by specific modification of cellulose features in transgenic rice
Source: Biotechnol Biofuels. 2019 Jan 9;12:11. doi: 10.1186/s13068-018-1351-1 (PMC6325865; doi:10.1186/s13068-018-1351-1)
Supplement: Supplementary file 1 — Additional file 1: Figure S1. Alignment between OsGH9B1 and OsGH9B3. Table S1. The primers used for gene cloning and expression analysis in this study. [file 13068_2018_1351_MOESM1_ESM.ppt]

## Slide 1
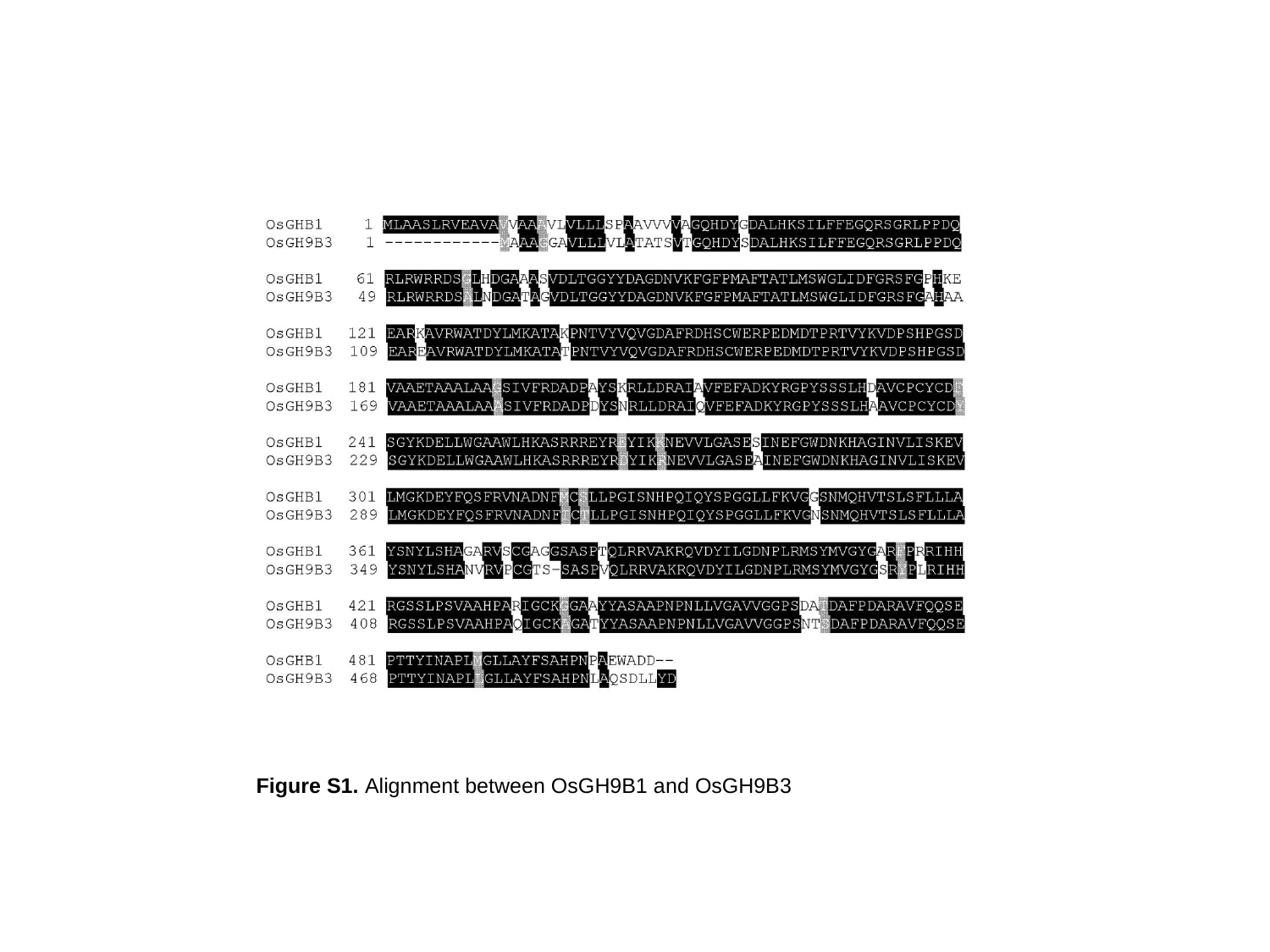

Figure S1. Alignment between OsGH9B1 and OsGH9B3

## Slide 2
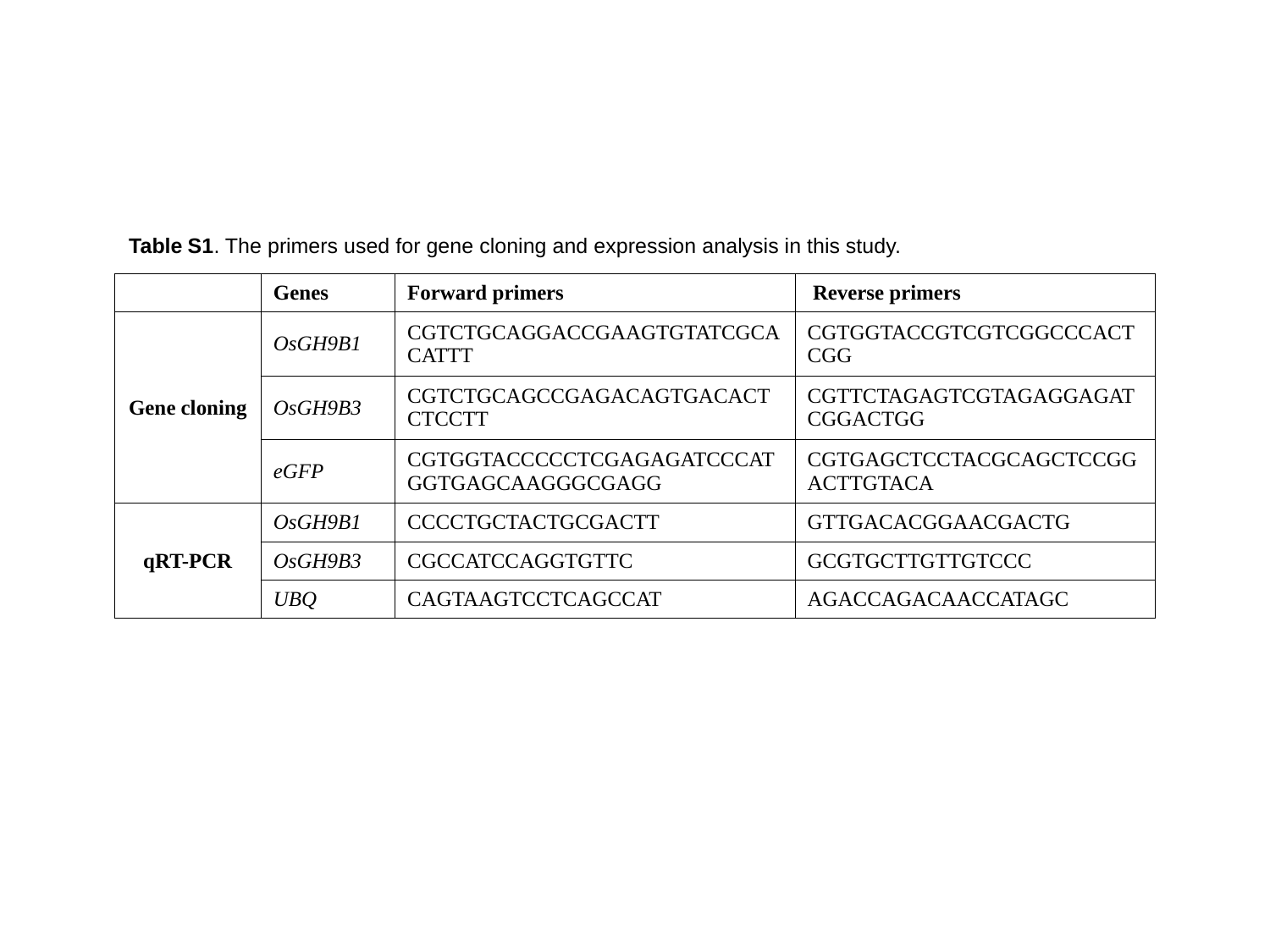

Table S1. The primers used for gene cloning and expression analysis in this study.
| | Genes | Forward primers | Reverse primers |
| --- | --- | --- | --- |
| Gene cloning | OsGH9B1 | CGTCTGCAGGACCGAAGTGTATCGCACATTT | CGTGGTACCGTCGTCGGCCCACTCGG |
| | OsGH9B3 | CGTCTGCAGCCGAGACAGTGACACTCTCCTT | CGTTCTAGAGTCGTAGAGGAGATCGGACTGG |
| | eGFP | CGTGGTACCCCCTCGAGAGATCCCATGGTGAGCAAGGGCGAGG | CGTGAGCTCCTACGCAGCTCCGGACTTGTACA |
| qRT-PCR | OsGH9B1 | CCCCTGCTACTGCGACTT | GTTGACACGGAACGACTG |
| | OsGH9B3 | CGCCATCCAGGTGTTC | GCGTGCTTGTTGTCCC |
| | UBQ | CAGTAAGTCCTCAGCCAT | AGACCAGACAACCATAGC |
